# Using High-Field Magnetic Resonance Imaging to Estimate Distensibility of the Middle Cerebral Artery

**DOI:** 10.1159/000446397

**Published:** 2016-07-23

**Authors:** Esther A.H. Warnert, Jasper Verbree, Richard G. Wise, Matthias J.P. van Osch

**Affiliations:** ^a^Cardiff University Brain Research Imaging Centre, School of Psychology, Cardiff University, Cardiff, UK; ^b^Department of Clinical Neurological Sciences, University of Western Ontario, London, Ont., Canada; ^c^C.J. Gorter Center for High-Field MRI, Department of Radiology, Leiden University Medical Center, Leiden, The Netherlands

**Keywords:** Arterial stiffness, Arterial structure/compliance, Cerebral small vessel disease, Middle cerebral artery, Ultra-high-field MRI

## Abstract

**Background:**

Although cerebral arterial stiffness may be an important marker for cerebrovascular health, there is not yet a measurement that accurately reflects the distensibility of major intracranial arteries. Herein, we aim to noninvasively measure distension of the human middle cerebral artery (MCA).

**Methods:**

Ten healthy volunteers (age: 30.3 ± 10.8 years) underwent ultra-high-field (7-tesla) MRI scanning. Time-of-flight angiography and phase-contrast flow imaging were used to locate the M1 segment of the MCA and to determine the occurrence of systole and diastole. High-resolution cross-sectional cardiac triggered T_2_-weighted images of the M1 segment of the MCA were acquired in systole and diastole.

**Results:**

The average distension of the MCA area from diastole to systole was 2.58% (range: 0.08%-6.48%). There was no significant correlation between MCA distension and the pulsatility index, calculated from the phase-contrast flow velocity profiles.

**Conclusion:**

These results lead to the first noninvasive image-based estimation of distensibility of the MCA (approx. 5.8 × 10^−4^ mm Hg^−1^) and demonstrate that ultra-high-field MRI could be a promising tool for investigating distensibility of intracranial arteries in relation to cerebrovascular pathology.

## Introduction

Healthy cerebral arteries are able to smooth out the pulsatile blood flow originating from the heart into an almost continuous flow into the capillary bed of the brain [1]. If the cerebral arteries stiffen, the pulsatile blood flow propagates further into the arterial tree, where the pulsatile shear stress can induce damage to the walls of the small vessels [2,3]. This process has been linked to severe pathologies, including cerebral small vessel disease and vascular cognitive decline [2,3], and highlights the potential value of a measure of cerebral arterial stiffness as a marker of cerebrovascular health.

Measuring stiffness (or its inverse: distensibility) in extracranial arteries is commonly done by assessing changes in diameter occurring with changes in pressure at the same site [4]. The skull complicates such measurements for intracranial arteries, which explains the absence of an accurate measurement of cerebral arterial stiffness. However, volume changes of approximately 5% occurring throughout the cardiac cycle in the middle cerebral artery (MCA) have recently been measured using computed tomography (CT) angiography [5].

In contrast to CT, magnetic resonance imaging (MRI) can be used noninvasively to measure the cross-sectional area of the MCA [6]. Based on the volume changes found by CT angiography [5], distention of the MCA during the cardiac cycle is expected to be small (0.05-0.1 mm). Noninvasive imaging of such small geometrical changes requires high-resolution measurements, which are currently only feasible with ultra-high-field MRI [6].

In this proof-of-principle experiment, we aimed to measure distension of the MCA by using ultra-high-field MRI and by synchronizing image acquisition to the cardiac cycle. In addition, we assessed the pulsatility index (PI) as a combined measure of cerebrovascular resistance and stiffness. To the best of our knowledge, this is the first noninvasive assessment of cerebral arterial distension in humans.

## Materials and Methods

Ten healthy participants were recruited for this experiment (6 females, all nonsmoking, average age 30.3 ± 10.8 years). Informed consent was obtained from all volunteers. This study was performed under approval of the Institutional Review Board of the Leiden University Medical Center according to the Declaration of Helsinki and in accordance with the guidelines for Good Clinical Practice (CPMP/ICH/135/95).

### Image Acquisition

**Fig. 1:**
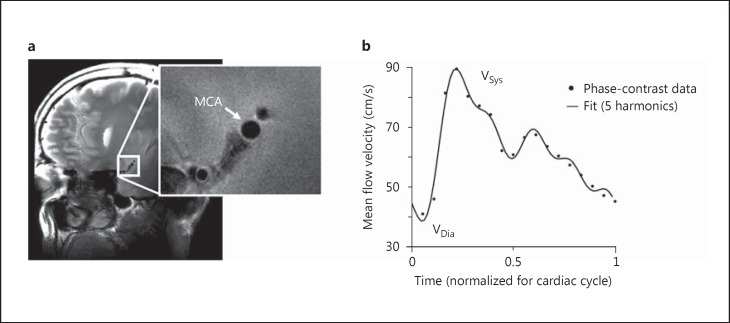
**a** Example of T_2_-weighted high-resolution structural image of the cross-sectional area of the M1 segment of the MCA. **b** Example of phase-contrast flow velocity data (dots) measured at the same location as in **a**. The flow velocity waveform was approximated by fitting the first five harmonics of a Fourier sequence (solid line), from which the pulsatility index was calculated. Sys = Systole; Dia = diastole.

MRI scans were performed at 7 T (whole-body Philips Achieva; Philips Healthcare, Best, The Netherlands) similar to a previously described protocol [6]. All image acquisition parameters are stated in the online supplementary data (for all online suppl. material, see www.karger.com/doi/10.1159/000446397). A 3-dimensional time-of-flight (TOF) angiogram was performed to identify the MCA and for planning of an imaging plane perpendicular to the M1 segment. Care was taken to select a straight portion of the MCA and exclude branching arteries. Planning of this imaging plane was guided by additional reconstructions of the TOF scan to better visualize the course of the MCA in all directions. The position and orientation of the imaging plane were copied to the quantitative flow scan to assess the flow velocity waveform through the MCA. Directly after acquisition, a circular region of interest in the center of the MCA was used to determine the flow velocity profile at the level of M1, which was used to determine the time points at which peak diastole and peak systole occurred and to calculate the post-trigger acquisition delay times for the cardiac triggered T_2_-weighted images. High-resolution and cardiac triggered T_2_-weighted images were acquired at 4 time points in the cardiac cycle in pseudo-random order: 200 (t_1_) and 100 ms (t_2_) preceding peak diastole, and 50 ms before (t_3_) and 50 ms after (t_4_) peak systole. These time points were chosen because in the carotid artery peak flow velocities have been shown to closely follow systolic and diastolic pressure [7]. An example of a high-resolution structural image can be seen in figure 1a. Throughout all scans, pulse oximetry at the finger was used to measure the cardiac pulse.

### Image Analysis

Two observers, blinded to participant and cardiac phase of the images, manually drew elliptical regions of interests on the T_2_-weighted images to delineate the internal wall of the MCA. Each observer repeated this process once, and the average of all 8 measurements (two scans per time point and two observers who delineated the MCA twice) per time point was used to calculate MCA cross-sectional area. Paired t tests were used to investigate whether the MCA area at time points t_1_, t_3_, and t_4_ were significantly different than at t_2_ (triggered closest to peak-diastolic velocity).

To investigate the consistency within and between observers, the intraclass coefficient of correlation for consistency [ICC(C,1), ICC(C,k)] was calculated with MATLAB (R2012b; MathWorks, Natick, Mass., USA).

The quantitative flow scan was also used to calculate a commonly used cerebral arterial stiffness index based on the blood flow velocity waveform through the MCA, PI [8,9]. A linear regression was performed (*robustfit* in MATLAB) between the distention of the MCA between peak diastole and peak systole (t_2_ and t_3_) and PI.

## Results

There was high consistency in MCA area measurements within observer [ICC(C,1)_Obs.1_ = 0.89, ICC(C,1)_Obs.2_ = 0.92] and between observers [ICC(C,k) = 0.91], justifying the averaging of all 8 MCA area measurements per time point.

**Fig. 2:**
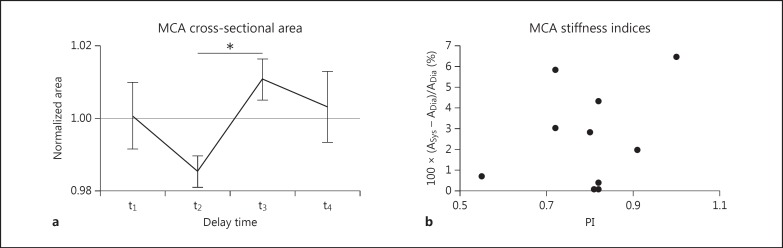
**a** Group average (n = 10) cross-sectional area of the MCA for 4 different delay times. Data were normalized per participant by dividing by the average MCA cross-sectional area of each individual. Note that t_2_ is the delay time closest to peak diastole and t_3_ the delay time closest to peak systole. There was a significant difference (* p < 0.01, paired t test) between the diastolic and systolic area. Error bars = SEM. **b** Increase in area from diastole (Dia) to systole (Sys) plotted against the PI for all 10 participants (no significant correlation, r^2^ = 0.13, p = 0.35).

There was a significant increase in MCA area between diastole and systole of 2.58% (min-max range: 0.08%-6.48%; t test, p < 0.01; fig. 2a).

The group average PI was 0.80 ± 0.12 and was not significantly correlated with the change in MCA area between systole and diastole (r^2^ = 0.13, p = 0.35; fig. 2b).

## Discussion

In the current study, it is shown for the first time that ultra-high-field MRI can be used to noninvasively measure distention of the MCA occurring through the cardiac cycle, which is a prerequisite for measurements of cerebral arterial stiffness. When combined with reference values for central pulse pressure in healthy participants (45 mm Hg [10]), the estimated average MCA distensibility is approximately 5.8 × 10^−4^ mm Hg^−1^.

This level of distensibility in the MCA is plausible, as the significant change in MCA area of 2.58 ± 2.4% through the cardiac cycle falls within the range of volume changes in the MCA measured with CT [5]. Furthermore, the resulting estimation of MCA distensibility follows the expectation that intracranial arteries are less distensible than extracranial arteries (reported distensibility in the carotid artery ranges from 0.5 × 10^−3^ to 5.8 × 10^−3^ mm Hg^−1^[4]).

There was no correlation between the distention of the MCA cross-sectional area and PI. Although in a small cohort, this finding highlights that in addition to local arterial stiffness, other factors such as stiffness and resistance of the downstream vascular bed shape the blood flow velocity waveform [11]. Furthermore, this illustrates the need for investigation of the relationship between cerebral arterial distensibility and the formation of blood flow velocity waveforms, which is an important step in understanding the mechanisms that link increased arterial stiffness to cerebrovascular pathologies, such as small vessel disease [9] and vascular cognitive decline [2]. Future studies investigating these mechanisms should include participants with a wide range of expected cerebral arterial distensibilities, e.g. young and elderly individuals or patients with small vessel disease.

A limitation of this study is that no blood pressure measurements were included, and, therefore, only an estimation of MCA distensibility was feasible. However, underlying pulse pressures are not expected to show large deviations from reference values [10], because only healthy and nonsmoking volunteers were recruited. Future work should include blood pressure measurements such that cerebral arterial stiffness can be quantified in terms of distensibility or compliance [12]. Although local pulse pressure measurements would be a requirement for accurate estimation of arterial distensibility [4], we recommend noninvasive measurements of blood pressure (e.g. brachial blood pressure [12]) in studies for which it is not feasible to invasively assess intracranial blood pressure.

In summary, we have shown that ultra-high-field MRI can be used to noninvasively measure cerebral arterial distensibility, and that this is a promising tool for future research into the relationship between cerebral arterial stiffness and cerebrovascular pathology.

## Disclosure Statement

The authors have no conflict of interest to disclose.
